# The Relationship between Happiness and “Deadly Sins” among Middle-Aged Persons

**DOI:** 10.11621/pir.2021.0315

**Published:** 2021-09-30

**Authors:** Margarita E. Permiakova, Olga S. Vindeker

**Affiliations:** Ural Federal University, Ekaterinburg, Russia

**Keywords:** Happiness, life satisfaction, “deadly sins;” middle age, sociodemographic factors

## Abstract

**Background:**

The contradictory results of studies on the relationship of happiness and well-being to norm-prohibitions make further work on this subject urgent. This topic is of particular relevance in connection with the current crisis of the value system.

**Objective:**

Our research was devoted to the study of happiness, life satisfaction, and compliance with norm-prohibitions in middle-aged Russians. We hypothesized that happiness is associated not only with life satisfaction but also with the ability to resist temptations (such as what are known as “mortal sins”). The survey used six temptations: wrath, greed, envy, sloth, gluttony, and extra pride. Resistance to these “sins” represented adherence to “norm-prohibitions”.

**Design:**

The study involved 1,520 respondents (222 male and 1,298 female). The mean age of the participants was 40.37 ± 6.01 years. The socio-demographic questionnaire included items related to gender, age, marital status, number of children, level of education, and financial situation. Happiness, life satisfaction, and adherence to “norm-prohibitions” were measured on a 10-point scale.

**Results:**

Happiness was associated with marital status, the number of children, and income per family member. It also correlated with life satisfaction, mostly in the area of relationships. Both men and women felt equally happy. The happiest people were less likely to manifest the “deadly sins” of wrath, greed, envy, and sloth. At the same time, happiness, calmness, and optimism were positively associated with pronounced gluttony and extra pride.

**Conclusion:**

The results indicate that a significant contribution to happiness is made by the ability of a person to adhere to norm-prohibitions.

## Introduction

The problem of human happiness is being actively investigated in the framework of positive psychology. In most languages of the world, there are words for happiness, but so far, there is no consensus among scientists about its essential meaning (Leontiev & Rasskazova, 2006; Dzhidaryan, 2013; Moyano Diaz, Dinamarca, Mendoza-Llanos, & Palomo-Vélez, 2018). On the one hand, its interpretation is close to the hedonic approach (Argyle, 2003; Diener, 2020; Seligman, 2006), according to which happiness is subjective well-being. In this sense, happiness includes cognitive (life satisfaction) and affective (balance of positive and negative emotions) components. On the other hand, according to the eudaimonic approach (Keyes & Waterman, 2003; Robinson & Ryff, 1999), the concept of happiness is closer to psychological well-being. In this case, happiness is an indicator of the positive functioning of the personality and is associated with self-actualization.

There is an active discussion between the proponents of the hedonic and eudaimonic approaches, and ideas expressed about the possibility and productivity of their integration (Demenev, 2016; Rikel, 2017; Sozontov, 2006; etc.). In that context, Dmitry Leontiev (2020) has offered a Two-level Model of Happiness. The first level is deficit (passive) happiness, which reflects the measure of satisfaction of basic needs and has a saturation point. The second one is self-deterministic (active) happiness; it is the subjective experience of achieving meaning and one’s chosen goals. At the same time, one feels and evaluates one’s level of happiness as a holistic experience.

Empirical studies of happiness have been devoted to research on its predictors and correlates. For each person, happiness is determined by his or her peculiar combination, since there are no universal recipes for happiness. Sources of happiness can be internal and external. External factors are socio-economic, ecological, and ethnocultural conditions that characterize the human environment. Internal factors of happiness include personal characteristics such as gender, age, temperament, character, family, satisfaction with one’s financial situation, work, interpersonal relationships, availability of free time for leisure, hobbies, success, self-actualization, values, faith, etc. (Leontiev & Rasskazova, 2006).

Studies have shown that feeling happy —high subjective well-being —leads to better health and increases life expectancy. Happy people rate their health level higher, regardless of the objective indicators. Physiological responses to stress among happy people are less pronounced (Diener & Chan, 2011; Eddington & Shuman, 2006). The hallmarks of happy people are optimism, high self-esteem, and a sense of personal control (Myers & Diener, 1995; Seligman, 2006). Happiness is negatively correlated with depression and anxiety. Studies on the Big Five personality traits have shown that happiness is negatively associated with neuroticism, and positively with extraversion and openness to new experiences (Diener & Seligman, 2002).

M. Csikszentmihalyi’s experience sampling method (ESM) revealed that happy people feel involved in their activities and are satisfied with them (Myers & Diener, 1995). They use productive coping strategies to deal with life’s challenges (Dzhidaryan, 2013). Less important for the feeling of happiness are material security, gender, and education (Eddington & Shuman, 2006; Myers & Diener, 1995). As for religious faith, there is no consensus on its significance. For example, Myers and Diener (1995) and Seligman (2006) found faith to be a significant factor. Religiously active people were less susceptible to psychological discomfort and stress, lived longer, and on average were happier than those who did not adhere to any religion. However, there is also an opinion that faith has little effect on the feeling of happiness (Argyle, 2003; Permiakova & Ershova, 2015).

Blanchflower (2020) studied the relationship between happiness and age in 145 countries, and confirmed the existence of a U-shaped age-of-happiness curve. The least happy people were around the age of 50. Based on the analysis of data from the European social survey (ESS) on the level of happiness and satisfaction with various aspects of life in 25 countries, Monusova (2012) concluded that Russia has the sharpest decline in subjective well-being with age of all the European countries.

At the same time, in all age groups, indicators of emotional assessment (happiness) are higher than from a rational assessment (life satisfaction). A small rise was observed only from 55 to 64 years, and after 65 years of age happiness declined. Such a non-linear tendency is due to the non-identical contribution of various partial satisfaction indicators to the integer index of subjective well-being in different age periods. For example, Russians are less satisfied with their financial situation, especially at the age of 45–50 years. At the same time, their job satisfaction increases dramatically after the age of 50. Young people look at life more optimistically than older generations of Russians, who have one of the lowest rates of optimism in Europe. Satisfaction with oneself and one’s health declines steadily with aging.

### Background

Despite the rather deep development of the described problems, the most studied component of happiness is life satisfaction (Temiz, 2020). At the same time, there has been very little research on the connection between happiness and compliance with human universal norms. Baumeister and Exline (1999) noted that psychology has always sought the scientific ideal, and tried not to make value judgments. This may have hindered the study of “virtue” and “sin.” According to Poddiakov (2012), in positive psychology it is quite rare to find an explicitly formulated attitude toward the problems of good and evil. Seligman (2006) described six common virtues for all peoples, but argued that science should take a neutral position in relation to morality, since the task of science is to describe, not prescribe. According to him, happiness implies the spiritual satisfaction of realizing one’s individual virtues and using them to serve a higher purpose.

A number of authors have pointed to a positive relationship between happiness and moral values. Philips, Freitas, Mott, Gruber, and Knobe (2017) noted that the concept of happiness is related not only to the components of subjective well-being but also to moral values. According to Bloomfield (2015), morality is necessary for self-esteem, and self-respect is necessary for happiness; therefore, morality is necessary for happiness. Waytz and Hofmann (2020) experimentally proved that ethical behavior and thoughts strengthen self-concept and empathy. Moreover, they are effective means of increasing subjective well-being. Data from a study by Deb, Thomas, Bose, and Aswathy (2020) showed that there was a significant positive correlation between spirituality and happiness.

Today, Russia is still working out ways of studying value orientations in the context of happiness and well-being. At the same time, the results of a few studies on the subject have been contradictory. Muravyova and Popkova (2010) investigated the relationship between subjective well-being and value orientations in students and found that emotional well-being was higher in those young people who focused on the values of understanding, tolerance, and protection of humanity and nature. Beskova (2015) showed that psychological well-being was associated with an orientation towards good/evil and humanity. The stronger a person was oriented towards evil, the more he felt his trouble subjectively. But at the same time, there was no significant connection with the orientation toward good.

Nekhorosheva (2012) found that people with a negative orientation (for example, selfishness, plagiarism, bribery) were more satisfied with their lives, while people with a positive orientation were less happy. Bocharova (2016) investigated ethnic features of subjective well-being of young people in samples of respondents of Armenian and Russian nationality. The results suggested a link between subjective well-being and morality in both samples, but the predictors differed. Semenova (2014) determined that psychological well-being positively correlated with religious self-awareness, with faith in God. At the same time, Permiakova and Ershova (2015) found no differences in the level of happiness between orthodox believers and atheists.

Shcherbatykh (2010) developed the concept of a moral norm, discussing both general aspects of morality and specific moral codes (for example, religious ones). A norm is a set of rules and regulations that determines human behavior in all spheres of life (family, interpersonal relationships, professional activities, and so on), and is aimed at achieving the good for oneself and others. Norms are in the form of a social contract. Society evaluates the “rightness” or “wrongness” of an individual’s behavior. At the same time, compliance with these norms is the individual person’s choice. Yao (2015) considered morality a special form of social value, which contains criteria for evaluating good and evil, and is aimed at making people happy.

There are different classifications of norms, including their division into norm-prohibitions (undesirable norms of behavior for society, immoral tendencies, or vices) and norm-commands (positive, desirable norms of human behavior, or virtues). Historically, norm-prohibitions have always preceded the rules-commandments. Each generation and community of people has developed its own understanding of good and evil, “virtues” and “vices.” Useful norms are fixed and passed down from generation to generation, becoming social attitudes and part of individual consciousness. Some of them have emerged within the framework of a religious concept and gradually became universal, because, despite cultural differences, most societies have common goals in terms of security, justice, and harmony. Such norms and prohibitions include the “seven mortal sins” from the Christian doctrine: pride, wrath, greed, envy, sloth, lust, and gluttony. They are called deadly because, according to religious beliefs, they lead to the destruction of the soul. The concepts of sin and virtue have been considered unscientific, but religious. Nevertheless, over the last two decades, they have become the object of research in psychological science (Shcher-batykh, 2010).

In religion, these sins are evaluated based on the dichotomy between good and evil, and are clearly considered bad and unworthy. In psychology, both the negative and positive aspects of sin are discussed: Examples include Shcherbatykh (2010) in the book *The Seven Deadly Sins, or the Psychology of Vice for believers and non-believers*; Ilyin (2014) in the book *Psychology of Envy, Hostility, and Vanity*, and Laham (2012) in the book *The Joy of Sin: Psychology of the Seven Deadly Sins.* Laham argued that these sins are not only beneficial but can also make a person successful and happy. The psychological definition of the essence of sin was based on the sin’s religious content, but each author introduced his own aspects, mainly due to linguistic and cultural differences.

The “seven deadly sins” are well known in everyday consciousness, but they are also described in the scientific literature (Barkley, Barkley, Curtis, & Hatvany, 2018; Laham, 2012; Shcherbatykh, 2014; Veselka, Giammarco, & Veron, 2014). Wrath includes excessive feelings of rage and a desire for revenge directed at people who offend or harm. Greed implies an excessive desire for money and material values. Envy arises because of the comparison with people who are more successful. Sloth is the emotional state when a person assesses his or her situation as almost hopeless and the outcome as inevitably unfavorable, and either hesitates or does not make efforts to get out of it. Lust is characterized by an excessive desire for sexual satisfaction. Gluttony is the excessive consumption of food and alcohol. Extra pride is expressed in vanity and excessive self-admiration. According to Laham (2012) and Shcherbatykh (2014), all of these temptations have both negative and positive aspects.

Khvostov and Gadzhimuradova (2016) showed that pride, wrath, greed, envy, sloth, lust, and gluttony are condemned by the majority of modern young Russians, regardless of gender, nationality, religion, or atheistic views. The differences related only to the degree of condemnation of each sin, depending on the person’s ethnic and cultural affiliation. In most empirical psychological studies, the “seven deadly sins” are used in their negative meaning. Thus, Barkley et al. (2018) investigated the relationship between self-control and the resistance to temptation. According to the authors, the most common temptations experienced by people are the “seven deadly sins,” which have stood the test of time and represent a powerful and useful taxonomy. Baumeister and Exline (2001) analyzed vice, sin, and virtue from the perspective of self-control theory. In their opinion, self-control is the main virtue because it allows overcoming antisocial urges and sinful behavior.

Veselka et al. (2014) investigated the relationship of subclinical forms of socially aggressive behavior, namely, the traits of the Dark Triad (Machiavellianism, narcissism, and psychopathy) with the seven deadly sins. All the Dark Triad traits were associated with major sins. The exception was a weak relationship between narcissism and sloth. Vrabel, Zeigler, McCabe, and Baker (2019) studied the links between the pathological personality traits included in the DSM-5 (negative affectivity, alienation, antagonism, disinhibition, and psychoticism) and the seven deadly sins. According to the authors, mortal sins are a taxonomy of destructive and self-destructive behaviors. People who do not follow moral norms (“who sin”) are prone to selfish, aggressive, and antagonistic thoughts and behavior.

Permiakova, Glinskikh, and Ershova (2018) investigated the relationship of happiness and psychological well-being with the observance of norm-prohibitions defined by the seven deadly sins in a sample of students. Contradictory data was obtained: namely, they showed a positive relationship with a sense of happiness and a negative correlation with psychological well-being. Thus, in psychology, there is still no clear answer to the question of whether happiness and psychological well-being are connected with the adherence to specific norm-prohibitions.

### The Current Study

The aim of our study was to determine how the violation of norm-prohibitions is associated with the feeling of happiness in people of middle age. This is the period of a person’s greatest working capacity. It is at this age that there is a steady decline in the level of subjective well-being. What we have added is the study of the relationship between happiness and compliance with norm-prohibitions as defined by the “deadly sins”. We hypothesized that happiness is negatively associated with such “deadly sins” as wrath, greed, gluttony, envy, extra pride, and sloth. Additionally, the relationship between happiness and life satisfaction was studied.

## Methods

### Participants

The study was conducted in 2018. It included 1,520 people (222 men and 1,298 women). The sample consisted of adults with one or more children. The mean age of the participants was 40.37 ± 6.01 years. *Table 1* describes the sample*.*

**Table 1:** Description of the Sample

Sample’s Parameters	Total	Female	Male
Respondents	1520	1298	222
	*Marital Status*		
Married	1165	969	196
Not married	355	329	26
	*Number of Children*		
One	556	483	73
Two	754	647	107
Three and more	210	168	42
	*Housing Conditions*		
Owner	1369	1174	195
Renting	151	124	27
	*Education*		
Secondary	476	398	78
Higher	1034	895	139

### Measures

The respondents were given a questionnaire that included points of a socio-demographic and psychological nature. The socio-demographic questionnaire included items related to gender, age, marital status, number of children, level of education, and financial status (*Table 1*).

All psychological parameters were assessed using a 10-point system, based on previously obtained data on the validity of the indicators, which are an alternative to “cumbersome” methods (Permiakova et al., 2018). The psychological parameters were:

1. *Life satisfaction.* The subjects used a 10-point scale (1 = the minimum level of satisfaction; 10 = the maximum level) to assess their level of satisfaction with the following aspects of their lives: work; health; financial situation; relationships with children, friends, and partner; and the opportunity for getting a good rest.
2. *Happiness.* The subjects rated how happy they were on a 10-point scale (1 = absolutely unhappy; 10 = absolutely happy). Additionally, the subjects assessed their level of optimism (1 = absolute pessimist; 10 = absolute optimist) and anxiety (1 = maximum level of anxiety; 10 = minimum).
3. *“Deadly sins.”* To determine the degree of observance of norm-prohibitions, the participants evaluated on a 10-point scale the level of their greed, wrath, envy, and sloth (1 = maximum; 10 = minimum level of sinfulness), as well as the severity of their gluttony and extra pride (1 = minimum; 10 = maximum level). The exception was “lust,” which was not included in the assessed parameters due to the context (given within the framework of parent meetings of secondary schools).

### Procedure

The survey of respondents was timed to coincide with parent-teacher conferences held in 12 secondary schools in Ekaterinburg (Ural Region, Russia). The study participants were parents who attended a parent-teacher meeting. All subjects were informed about the purpose of the study, and gave their voluntary consent to participate. As an incentive to participate, subjects were given the opportunity to receive free psychological counseling on any problems affecting their children’s educational success. The study was anonymous, and the participants were informed about the conditions of confidentiality.

### Data Analysis

Statistical analysis included Fisher’s ϕ^*-^angular transform; ANOVA analysis; Pearson’s correlation coefficient; and Factor Analysis (Principal Components, Varimax Normalized).

## Results and Discussion

### Comparative Analysis

A comparison of the percentage of men and women with different levels of happiness, life satisfaction, and compliance with norms-prohibitions showed the following. Among men, the percentage of those who were completely satisfied with their marital relations (60.4 %; ϕ^*^ = 2.18, p < 0.05) and the possibility of full rest (27.5%; ϕ^*^ = 2.64, p ≤ 0.01) was significantly higher than among women (52.5 and 19.4%, respectively). Among men, there were more respondents with a low level of satisfaction with their relationships with friends compared to women (5.9 vs. 2.7%; ϕ^*^ = 2.21, p ≤ 0.05).

Although the level of happiness and optimism of respondents was evenly distributed regardless of gender, men were a little more anxious (14.4%; ϕ^*^ = 1.94, p ≤ 0.05) than women (9.8%). There were more women who failed to abstain from greed (29.0%; ϕ^*^ = 1.74, p ≤ 0.05) than men (23.4%). However, among men, there were more respondents who could not resist extra pride (49.6 vs. 42.9%; ϕ^*^ = 1.85, p ≤ 0.05). Men were significantly more resilient (6.3 vs. 3.0%; ϕ^*^ = 1.74, p ≤ 0.05). Greater numbers of men than women were satisfied with their financial situation, marital relations, and the possibility of getting a full rest.

*Table 2* presents the results of variance analysis for all the variables in the groups of women and men, as well as in the groups of respondents who were married and not married (one-factor analysis separately for gender and marital status).

**Table 2:** Results of one-factor analysis of variance (ANOVA)*

	Scales	Gender				Marital Status			
		Ma	Fe	F9MII	ft	Mr	nMr	F	ft
Life Satisfaction Areas	Financial Situation	6.77	6.41	4.77	0.03	6.63	5.91	29.30	0.00
	Partner’s Relationship	8.21	7.76	5.92	0.02	8.43	5.82	350.46	0.00
	Relationship with Children	8.38	8.54	1.55	0.21	8.64	8.10	25.91	0.00
	Friends’ Relationship	8.15	8.42	3.79	0.05	8.47	8.06	13.42	0.00
	Opportunity of Full Rest	6.46	6.24	1.46	0.23	6.40	5.85	13.83	0.00
	Health	7.08	6.94	0.95	0.33	7.03	6.72	6.64	0.01
Well being	Happiness	7.77	7.97	1.95	0.16	8.03	7.67	9.12	0.00
	Anxiety – Calm	3.55	3.92	5.86	0.02	3.80	4.07	4.94	0.03
Resistance to greed		3.28	2.93	4.45	0.04	2.99	2.97	0.01	0.91

*Note. Ma = male, Fe = female; Mr = married, nMr = not married.*

*Table 2* shows that gender and marital status affected some parameters of life satisfaction, as well as the level of happiness and anxiety. At the same time, these factors did not affect adherence to norm-prohibitions, with the exception of resistance to greed. Men had a higher level of satisfaction with their financial situation and marital relations; they had higher anxiety and were more resistant to greed. Married persons were more satisfied with their financial situations, relationships with children, spouses, and friends, as well as being more satisfied with their health and the possibility of getting a full rest. In general, married people were happier, irrespective of gender.

### Correlation Analysis

Initially, it appeared that the feeling of happiness closely correlated with calm and optimism. We have combined all these parameters into the “well-being” group. Correlation analysis confirmed a close relationship between happiness and calmness (r = 0.39) and optimism (r = 0.549). A significant correlation between the parameters of well-being and life satisfaction with various aspects of life, compliance with norm-prohibitions, and some socio-demographic indicators is presented in *Table 3.*

**Table 3:** Happiness, life satisfaction, and norms-prohibitions scales

		Well-being Parameters		
	Parameters	Happiness	Calm	Optimism
Life Satisfaction Areas	Job	.39^***^	.29^***^	.28^***^
	Financial Situation	.38^***^	.31^***^	.26^***^
	Partner’s Relationship	.39^***^	.21^***^	.22^***^
	Relationship with Children	.48^***^	.28^***^	.31^***^
	Friends’ Relationship	.47^***^	.23^***^	.34^***^
	Health	.38^***^	.32^***^	.26^***^
	Opportunity of Full Rest	.38^***^	.32^***^	.31^***^
Resistance to…	…wrath	.43^***^	.47^***^	.58^***^
	…greed	.41^***^	.26^***^	.50^***^
	…envy	.43^***^	.29^***^	.48^***^
	…sloth	.53^***^	.35^***^	.55^***^
Gluttony		.16^***^	.16^***^	.17^***^
Extra Pride		.51^***^	.37^***^	.55^***^
Socio demographic Factors	Age	–.09^*^	–.02	–.04
	Income per Family Member	.12^**^	.12^**^	.06^*^
	Number of Children per Family	.13^***^	.04	.10^*^
	Marital Status	.07^*^	.06^*^	–.01

Note. ^*^p ≤ .05; ^**^p ≤ .01; ^***^p ≤ .001.

Correlation analysis revealed a positive relationship of happiness with all indicators of satisfaction with various aspects of life, as well as with optimism and calmness. These results correspond to numerous empirical data obtained in similar studies. Happiness was positively associated with such socio-demographic indicators as income per family member, the number of children, and positive marital status, and did not depend on education and the availability of comfortable housing. The only negative correlation was found between happiness and the age of the respondents: the closer the respondents were to the age of 50, the lower the level of happiness. At the same time, age was not associated with calmness and optimism.

Positive associations of well-being parameters with compliance with such norm-prohibitions as wrath, greed, envy, and sloth were observed. However, at the same time, happiness, calmness, and optimism were associated with pronounced gluttony and extra pride.

### Factor Analysis

**Figure 1.**
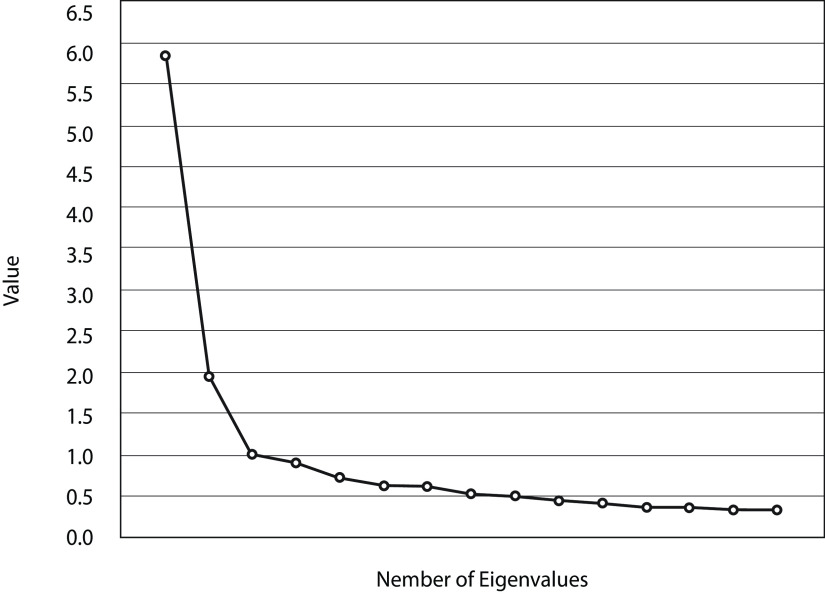
The Plot of Eigenvalues

Factor analysis of the indicators of “norm-prohibitions,” life satisfaction, and happiness parameters was performed using principal components analysis. According to the scree plot, three factors were identified: Factor I, Eigen. = 5.85, Var. Expl. = 0.40; Factor II, Eigen. = 1.95, Var. Expl. = 0.13; and Factor III, Eigen. = 1.01, Var. Expl. = 0.06) (see *Figure 1*).

Next, the procedure of normalized rotation of the three factors was performed (Varimax Normalized). As a result of rotation, the following results were obtained: Factor I, Eigen. = 4.24, Var. Expl. = 0.26; Factor II, Eigen. = 2.96, Var. Expl. = 0.19; and Factor III, Eigen. = 2.11, Var. Expl. = 0.13). (See *Table 4.*)

**Table 4:** Results of factor analysis of life satisfaction, happiness, and norm-prohibitions scales (n = 1520)

	Parameters	Factor I	Factor II	Factor III
Life Satisfaction Areas	Job	.14	.72	.26
	Financial Situation	.08	.82	.20
	Partner’s Relationship	.10	.33	.65
	Relationship with Children	.23	.30	.74
	Friends’ Relationship	.25	.28	.72
	Health	.12	.73	.24
	Opportunity of Full Rest	.19	.68	.23
Well–being Parameters	Happiness	.57	.32	.42
	Anxiety – Calm	.48	.48	–.07
	Optimism	.74	.21	.14
Resistance to…	…wrath	.73	.22	.05
	…greed	.75	.01	.12
	…envy	.74	.02	.18
	…sloth	.76	.13	.18
Gluttony		.37	.22	–.30
Extra Pride		.77	.15	.15
Eigenvalues		4.24	2.96	2.11
Proportion of Explained Variance		.26	.19	.13

Factor I consisted of norm-prohibitions’ scales, while the second and third factors measured satisfaction with various aspects of life. This factor structure indicated the relative independence of these aspects of life. At the same time, life satisfaction was not homogeneous. It clearly differentiated into the areas of physical, material, and business satisfaction on the one hand (II Factor Eigen. = 2.96; Var. Expl. = 0.19), and the sphere of personal relations (III Factor Eigen. = 2.11; Var. Expl. = 0.13) on the other. It is noteworthy that calmness (-0.07) and optimism (0.14) did not contribute to the relationship factor. As for happiness, it distributed among the three factors almost evenly, yet had a greater contribution (0.57) to the factor of norm-prohibitions.

Factor analysis conducted separately for pairs of descriptors “satisfaction/happiness” (Eigen. = 4.05, Var. Expl. = 0.51), and “norm-prohibitions/happiness” (Eigen. = 3.63, Var. Expl. = 0.52), showed the following. In the first case, the Happiness scale received a high load (0.68), but was insufficient in comparison with the satisfaction scales in various spheres of life. For comparison, see the following loads: satisfaction with job (0.73); financial situation (0.75); marital relations (0.66); relationships with children (0.74) and friends (0.72); the possibility of full rest (0.72); and health (0.70). This result confirmed our assumption that satisfaction, although an important factor in happiness, does not completely determine it. On the contrary, in the factor analysis of the scales of norm-prohibitions and the level of happiness, the Happiness scale received a high load (0.71). For comparison, see the following loads: resistance to “sinful manifestations” of wrath (0.74); greed (0.75); envy (0.76); and sloth (0.81). At the same time, happiness (0.71) did not exclude extra pride (0.81) and a slight degree of gluttony (0.35).

## Discussion

Our study of happiness levels showed that the majority of respondents of middle age (83.3%) rated their level of happiness as high. This does not contradict the data of the happiness level monitoring conducted by the Russian Center for the Study of Public Opinion in 2018, according to which 84 of respondents called themselves rather happy. On the other hand, based on the U-shape of the curve of the relationship of happiness with age (Monusova, 2012), the level of happiness obtained in the study was considerably higher than the average for this age group of Russians. Perhaps this is due to the fact that the study was conducted among the inhabitants of the Urals. According to a sociological study, they are the happiest Russians (Smoleva, 2020).

A person evaluates his or her level of happiness as a holistic experience, but behind this feeling is an individual combination of satisfaction with various areas of life, and a tendency toward more frequent manifestation of positive or negative emotions. These cognitive and affective components of happiness have been studied in numerous cross-cultural research projects. Our results once again confirmed the repeatedly proven data on the positive relationship of happiness with optimism and low anxiety, as well as with satisfaction with the most important areas of life: job, financial situation, health, and relationships with children, partner, friends, and the possibility of full rest.

However, men were more satisfied with their financial situation than women. This has objective causes. According to the calculations of The Institute of Economics of the Russian Academy of Sciences, the ratio of women’s wages to men’s wages is currently 72.1 (RIA Novosti, 2020). More men were satisfied with their marital relationships, but they did not have enough communication with friends. Perhaps this was due to the men making fewer demands on women, and communication with friends being one of their main needs. In such communication, men’s desire for independence is realized and their masculinity is confirmed. In Russia, traditionally, domestic duties and raising children are carried out by women; women objectively have less time to communicate with friends, so they make higher demands on marital relations.

The main part of the study was devoted to the relationship of happiness with norm-prohibitions. This topic is most deeply developed within the framework of philosophy and religious concepts. Aristotle noted that happiness is the goal of human activity, and defined it as the activity of the soul according to virtue. Happiness requires both the fullness of life and the fullness of virtue (Paleev, 2018). Achieving happiness is always associated with the problem of choice, with a constant struggle between Good and Evil. Happiness consists of overcoming temptations and constant spiritual development (Korko & Fomenko, 2020; Lyubetsky & Samygin, 2019).

Therefore, we suggested that happiness might be associated with adherence to norm-prohibitions. Today’s universal norms and prohibitions include the “seven deadly sins.” Correlation analysis confirmed links between the happiness of Russians in middle age with all “sins:” negative with wrath, greed, envy, and sloth; positive with extra pride; and weak positive with gluttony. Permiakova et al. (2018) have obtained similar results on a sample of students ages 17 to 20 years. Among the respondents were people of different faiths. These data confirmed that norm-prohibitions (confronting deadly sins) are universal values. Perhaps, for modern respondents, gluttony and extra pride have a different meaning and do not contradict norms. This may be due to the values of a market society, where the theme of success and consumption comes first. This aspect requires additional research.

Three relatively independent factors that determine a person’s happiness were identified. These were satisfaction with relationships, material and business satisfaction, and compliance with norm-prohibitions. For happiness, the most important factor was adherence to norm-prohibitions. The parameters of satisfaction depend on external circumstances and living conditions, while compliance with norm-prohibitions is an internal factor. According to Laurie Santos, a professor at Yale University, there is no clear correlation between happiness and external circumstances. The way to happiness is to change yourself (Santos, 2018). Professional practice shows that the reasons for a large number of the psychological problems that come to psychologists, especially concerning relationships between people, are due to non-compliance with these norm-prohibitions. Everyone knows what is “good” and what is “bad”. The discrepancy between this knowledge and a person’s actual actions is the cause of internal and external conflicts, which inevitably reduce life satisfaction and happiness.

## Conclusion

1. Happiness connected with some socio-demographic factors. Those who were married and had more than one child felt happier. Married respondents showed a higher level of satisfaction in all areas of life. The tendency to adhere to social norm-prohibitions among respondents with children did not depend on whether they were married or not.
2. Happiness was associated with life satisfaction and was clearly divided into two areas – satisfaction with the vital, material side of life and satisfaction with relationships. Happiness closely related to satisfaction with relationships. Gender characteristics must also be taken into account: men were more satisfied with their financial situation and marital relations. At the same time, both men and women felt equally happy.
3. A high level of happiness was associated with an assessment of one’s own tendency to adhere to norm-prohibitions. The happiest people were less likely to report manifestations of “deadly sins” – wrath, greed, envy, and sloth. At the same time, happiness did not contradict gluttony and pride.

The results indicate that, at the level of representations and self-assessment, the ability of a person to adhere to norm-prohibitions makes a significant contribution to happiness. We focused on “mortal sins” (prohibitions) but did not study the “norm-decisions” (behest) that are relevant to human virtue. It is possible that virtues contribute even more to subjective well-being and happiness. We plan to explore the relationship of happiness not only with adherence to norm-prohibitions but also with norm-decisions and virtues in the future.

## Limitations

The study sample consisted of middle-aged persons permanently residing in the territory of Ekaterinburg (Ural region). The respondents who took part in the study were parents who attended parent-teacher conferences at school and expressed a desire to take the survey. Accordingly, the results cannot extend to childless people and those who live outside the Ural region. Moreover, the study included significantly more women than men and did not use a scale of social desirability.
